# Network analysis of gene expression reveals regulators of cell viscosity and mechanical phenotype

**DOI:** 10.1038/s41598-025-11698-0

**Published:** 2025-09-30

**Authors:** Katherine M. Young, Nicole Latka, Roman Mezencev, Todd Sulchek

**Affiliations:** 1 Department of Bioengineering, Rice University, 6100 Main St, Houston, TX 77005-1827 USA; 2 School of Biology, Georgia Institute of Technology, 313 Ferst Drive, Atlanta, GA 30332-0405 USA; 3 Wallace H. Coulter Department of Biomedical Engineering, Georgia Institute of Technology, 313 Ferst Drive, Atlanta, GA 30332-0535 USA; 4 George W. Woodruff School of Mechanical Engineering, Georgia Institute of Technology, 801 Ferst Drive, Atlanta, GA 30332-0405 USA

**Keywords:** Cell viscoelasticity, Network analysis, Molecular regulators, Ovarian cancer, Biological physics, Gynaecological cancer, Cell biology, Gene expression

## Abstract

Cell mechanical properties, such as cell stiffness and viscous behavior, have been proposed as biomarkers of cell disease states. Moreover, the molecular pathways that modify cell mechanics may also be potential novel targets for managing lethal diseases, such as cancer, by specifically changing the mechanical phenotype of cells along with the associated functional phenotype. This study explores the relationship between the viscosity and stiffness of cells and the underlying molecular mechanisms. We used a large linked molecular dataset to explore the correlations between gene expression, cell migration, and cell mechanical properties, which were quantified by two viscous rate constants from a standard linear solid viscoelasticity model and apparent Young’s modulus from a Hertzian contact mechanics model. Using a causal network analysis built on known relationships curated from literature in Qiagen’s Ingenuity Pathway Analysis package, we identified potential molecular control nodes that could modify the expression of multiple genes correlated with cell mechanics. We investigated the up- and down-regulation of expression by two predicted potential small molecule regulators (lacidipine and AG879) and four predicted potential gene regulators (*AKT2*, *ITGB6, mir-183,* and *CD82*) through small molecule inhibition, RNA interference, and introduction of microRNAs. The effects of modulation of these regulators were measured on both cell mechanical properties and gene expression in three ovarian cancer cell types. We identified several regulators that change the viscosity and stiffness of the cell with a corresponding change to the functional migratory ability in a cell-type specific manner.

## Introduction

Ovarian cancer is a lethal gynecological malignancy with low survival rates largely due to late-stage diagnoses. For example, between 1975 and 2021, at the time of diagnosis only 19% of ovarian cancer cases in the SEER 22 cancer registries were limited to the ovaries, while 74% showed advanced cancer spread to regional or distant sites (7% were unstaged)^1^. Late stage diagnoses result in low 5-year survival rates and reduced treatment options for many patients. As traditional cytotoxic chemotherapies show limitations in managing the disease, researchers have turned to other molecular agents to target new pathways associated with aberrant signaling in cell proliferation, migration, invasion, survival, apoptosis, and angiogenesis^2^. Because the intrinsic mechanical properties of cells affect both the cell’s responses to its microenvironment and the overall progression of multiple cancer types, with softer and less viscous cells often being associated with worse prognosis, cellular mechanics is of interest as a targetable disease biomarker that can be manipulated through several integrated signaling pathways^3–10^. While the specific role of the expression of a number of cytoskeletal proteins on cancer cell mechanics has been studied before^11–14^, many other gene and protein signaling pathways that affect cancer cell mechanics remain unknown.

In previous work, we developed a method to investigate the intersection between cell mechanotype, functional phenotype, and genotype^15^. The single cell genomechanics method we have previously described combines single cell mechanical measurements of cell viscous and stiffness properties determined by atomic force microscopy, micropipette aspiration for the isolation of specific individual cells, and multiplexed targeted single cell RT-qPCR of 85 genes of interest to correlate single cell gene expression with cell mechanical properties across ovarian cancer cell lines of different metastatic ability. Of these genes of interest, 53 genes were significantly correlated with cell mechanical properties, with 2 genes uniquely correlated with the cell’s fast viscous rate constant (λ_1_), 12 genes uniquely correlated with the cell’s slow viscous rate constant (λ_2_), 13 genes uniquely correlated with the cell’s apparent Young’s modulus, and 26 genes corelated with multiple mechanical properties (Supplementary Fig. 1). If we are able to identify control nodes in pathways that alter multiple genes correlated with mechanical properties that could link cell mechanics and metastatic ability, we ultimately could perturb these control nodes as therapeutic targets for better management or prevention of cell migration and metastatic disease.

The main focus of this manuscript is to test several hypotheses generated by the single cell genomechanical method to modify genes of interest and adjust targeted cell mechanical properties. Because decreased cell stiffness and decreased cell viscosity are both associated with ovarian cancer malignancy, we explore the genetic regulation of both mechanical properties. Through the use of a genomic network analysis, we have identified several putative control nodes that can regulate the expression of our previously identified genes significantly correlated with the fast viscous rate constant, the slow viscous rate constant, and cell stiffness. We chose to focus our study on 6 regulators predicted by the network analysis, four predicted potential gene regulators, *AKT2, ITGB6, miR183,* and *CD82,* and two predicted potential small molecule regulators, lacidipine and AG879, which represent both predicted activators and inhibitors of our three mechanical parameters of interest. To experimentally evaluate the effect of up- and down-regulation of these potential regulators, we used small molecule inhibition, RNA interference, and microRNA manipulation in multiple ovarian cancer cell lines while observing the changes in cell mechanical properties, gene expression, and cell migratory ability. This has led to the discovery of several regulators that modify cell mechanics and migration in a cell-type specific manner, including some of the first tied to cell viscosity.

## Results

### Network analysis results in hypothesis generation and testing of putative targets that affect cell viscosity

Given the wide range of genes previously identified in our genomechanical analysis that correlate with mechanical properties (Supplementary Fig. 1), we hypothesized that there are specific genes that can serve as cell mechanotype-related control points. We used the single cell correlation dataset generated in our recent work^15^ to identify potential control nodes that could regulate the expression of genes significantly correlated with cell viscous properties. We performed a network analysis (see Methods) of the genes significantly correlated with cell fast and slow viscous rate constants, λ_1_ and λ_2_, to identify potential upstream putative regulators that affect the expression of these genes. The analysis resulted in 30 and 39 significant predicted control nodes for λ_1_ and λ_2_, respectively, of which 22 were selected for representation because of their function as transcription factors, enzymes, kinases, growth factors, and other regulatory molecules (Table 1, Table 2, Supplementary Fig. 2, and Supplementary Fig. 3). Regulators with positive activation z-scores are associated with the increase of expression of genes positively correlated with λ_1_ or λ_2_ or the decrease of expression of genes negatively correlated with λ_1_ or λ_2_; regulators with negative activation z-scores should produce the opposite effect. By relying on functional annotations, we discovered genes positively correlated with λ_1_ were associated with the functional pathways of cell development and apoptosis while the negatively correlated λ_1_ genes were associated with cell proliferation. Genes positively correlated with λ_2_ were associated with cell movement, growth inhibition, and cell signaling, while the negatively correlated λ_2_ genes were associated with hematological cancers. To investigate whether the predicted regulators could affect the viscous properties as expected, we chose four regulators to test: *AKT2*, predicted to decrease λ_1_, lacidipine, predicted to increase λ_1_, AG879, predicted to decrease λ_2_, and *ITGB6*, predicted to increase λ_2_. (Table 3). These regulators were selected because they would allow us to test both activators and inhibitors of both λ_1_ and λ_2_ that could be easily perturbed through treatment with a small molecule drug or through RNA interference with a commercially available, tested siRNA that was predicted to influence many genes correlated with the viscous rate constant, potentially allowing these observed effects to be more clinically relevant. Additionally, these four regulators were predicted to perturb genes that were uniquely correlated with each viscous rate constant (such as *CTNNB1* and *TP53* for λ_1_ and *CDH3, COL5A1, FN1, LAMC2, LOX, MMP3, POSTN,* and *RB1* for λ_2_), potentially allowing for the discovery of regulators that are specific to each mechanical parameter of interest.

**Table 1:** Network analysis reveals control nodes that could control cell fast viscous rate constant (λ_1_).

λ_1_ **Control node regulators**	**Molecule type**	**Activation z-score**	**Network bias-corrected p-value**
**Lacidipine**	Small molecule	2.449	0.0038
PAX5	Transcription regulator	2.000	0.0006
CCN5	Growth inhibitor	2.000	0.0058
NDRG1	Enzyme	2.000	0.0080
**AKT2**	Kinase	−2.000	0.0640
MMP9	Enzyme	−2.000	0.0497
TBX3	Transcription regulator	−2.000	0.0378
WNT5A	Cytokine	−2.236	0.0190
CREB3	Transcription regulator	−2.646	0.0186

Network analysis of genes correlated with cell viscous rate constant, λ_1_, revealed 30 significant potential control nodes, 9 of which are listed here. Bolded potential regulators were experimentally tested.

**Table 2:** Network analysis reveals control nodes that could affect cell slow viscous rate constant (λ_2_).

λ_2_ **Control node regulators**	**Molecule type**	**Activation z-score**	**Network bias-corrected p-value**
ERBB3	Kinase	2.449	0.0143
YAP1	Transcription regulator	2.236	0.0464
**ITGB6**	Cell adhesion molecule	2.236	0.0316
WWTR1/TAZ	Transcription regulator	2.121	0.0267
mir-221	miRNA	2.111	0.0410
miR-17-5p	miRNA	−2.000	0.0167
SPDEF	Transcription regulator	−2.000	0.0219
CADM1	Cell adhesion molecule	−2.309	0.0169
mir-223	miRNA	−2.324	0.0300
**AG879**	Small molecule	−2.357	0.0142
TEAD2	Transcription regulator	−2.449	0.0162
CCN5	Growth inhibitor	−2.646	0.0002
Estrogen receptor	Transcription regulator	−2.714	0.0001

Network analysis of genes correlated with cell viscous rate constants, λ_2_, revealed 39 significant potential control nodes, 13 of which are listed here. Bolded potential regulators were experimentally tested.

**Table 3:** Selected control node regulators and how they were tested.

Control node regulators	Mechanical property affected	Predicted increase or decrease	Manipulation method	Expected change in property after manipulation
AKT2	λ_1_	Decrease	Small molecule inhibition with BAY1125976	Increase
Lacidipine	λ_1_	Increase	Small molecule drug: Lacidipine	Increase
AG879	λ_2_	Decrease	Small molecule drug: AG879	Decrease
ITGB6	λ_2_	Increase	RNA interference (siRNA) against ITGB6	Decrease
mir-183	Stiffness	Decrease	miRNA mimic – hsa-miR-183-5p	Decrease
CD82	Stiffness	Increase	Small molecule drug – 17-ODYA	Increase

The seven node regulators listed below were predicted to change the mechanical properties λ_1_, λ_2_, or cell stiffness. The manipulation method used to modify the control nodes is listed along with the predicted change in the mechanical property expected to be observed after manipulation.

**Fig. 1:**
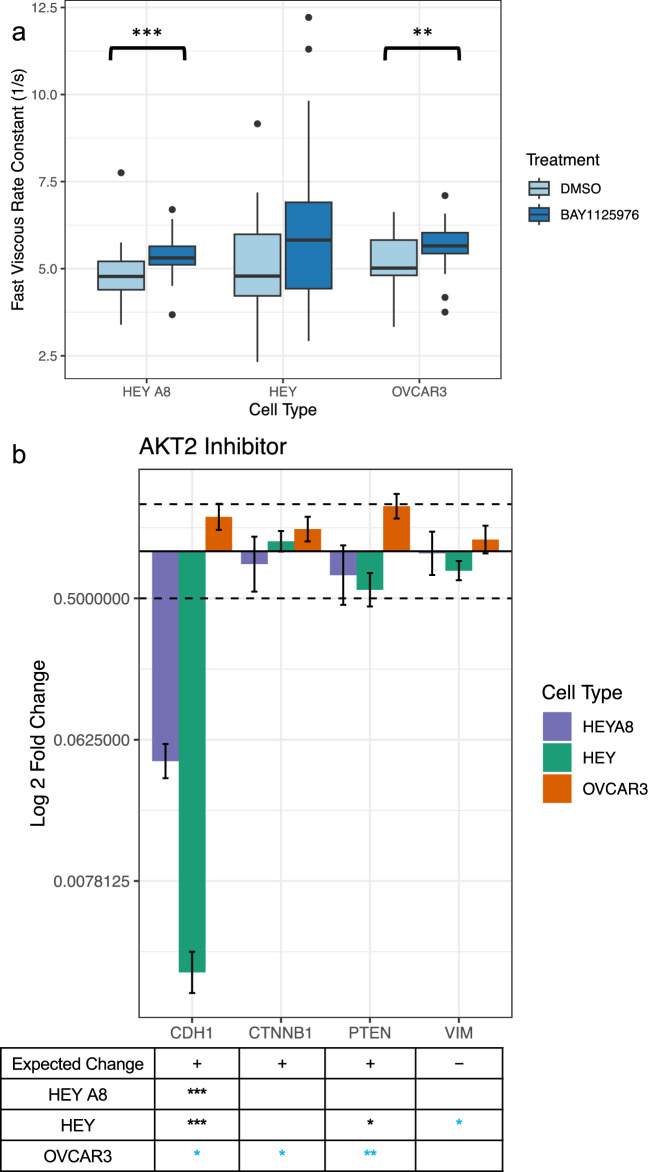
*AKT2* inhibition results in increase of λ_1_ as predicted **a**) Boxplots comparing the fast viscous rate constant, λ_1_, of cells treated with *AKT2* inhibitor BAY1125976 for 24 h. n = 40 for each treatment condition. *AKT2* inhibition significantly increased λ_1_ for HEY A8 and OVCAR3 cells (Welch two sample t-test, **, p < 0.01, ***, p < 0.001). **b**) Cells were treated with BAY1125976 and RNA was extracted from cells 24 h post-treatment. RT-qPCR was used to detect the fold change differences of expression of predicted gene targets when normalized to housekeeping genes *GAPDH* and *RPL32* and expression levels of cells treated with a vehicle negative control. The two dotted lines indicate a twofold expression change threshold increase and decrease. Error bars represent the standard deviation of PCR data after error propagation. The table below the bar plot indicates the expected change in gene expression as predicted by the network analysis with the number of stars representing significant p-values for changes in gene expression between the negative control and treatment group after housekeeping gene normalization (Welch’s two sample t-test, *, p < 0.05, **, p < 0.01, ***, p < 0.001). Blue stars indicate genes that significantly changed in expression that align with the predicted change.

The network analysis predicted a decrease in *AKT2* expression would result in an increase of fast viscous rate constant λ_1_. We treated mesenchymal highly-metastatic HEY A8, mesenchymal less-metastatic HEY, and epithelial least-metastatic OVCAR3 ovarian cancer cell lines with 10 µM of the small molecule chemical drug BAY1125976 for 24 h to induce *AKT2* inhibition^16^ followed by AFM measurement of the cell viscous properties. After *AKT2* inhibition, we observed an increase in λ_1_ as expected in all three cell lines (although the increase in the HEY cell line was not statistically significant) (Welch’s two sample t-test, HEY A8 – p < 0.001, HEY – p = 0.056, OVCAR3 – p < 0.01) (Fig. 1a). While there was significant increase of 3 of the 4 genes correlated with cell viscosity predicted to be associated with *AKT2* for the epithelial OVCAR3 cell line (Supplementary Fig. 2f), surprisingly we saw a large fold-expression decrease in *CDH1* in the mesenchymal cell types HEY A8 and HEY, a gene which was already lowly expressed in these cell lines prior to treatment (Fig. 1b). Changes to *CDH1*, a classical epithelial marker, along with a significant decrease of *VIM,* a mesenchymal marker, for the HEY cell line that aligns with the predicted network analysis, point to the importance of *AKT2* inhibition in a cell’s epithelial or mesenchymal state while our data highlights how changes to this state may also be associated with cell viscous relaxation rates.

**Fig. 2:**
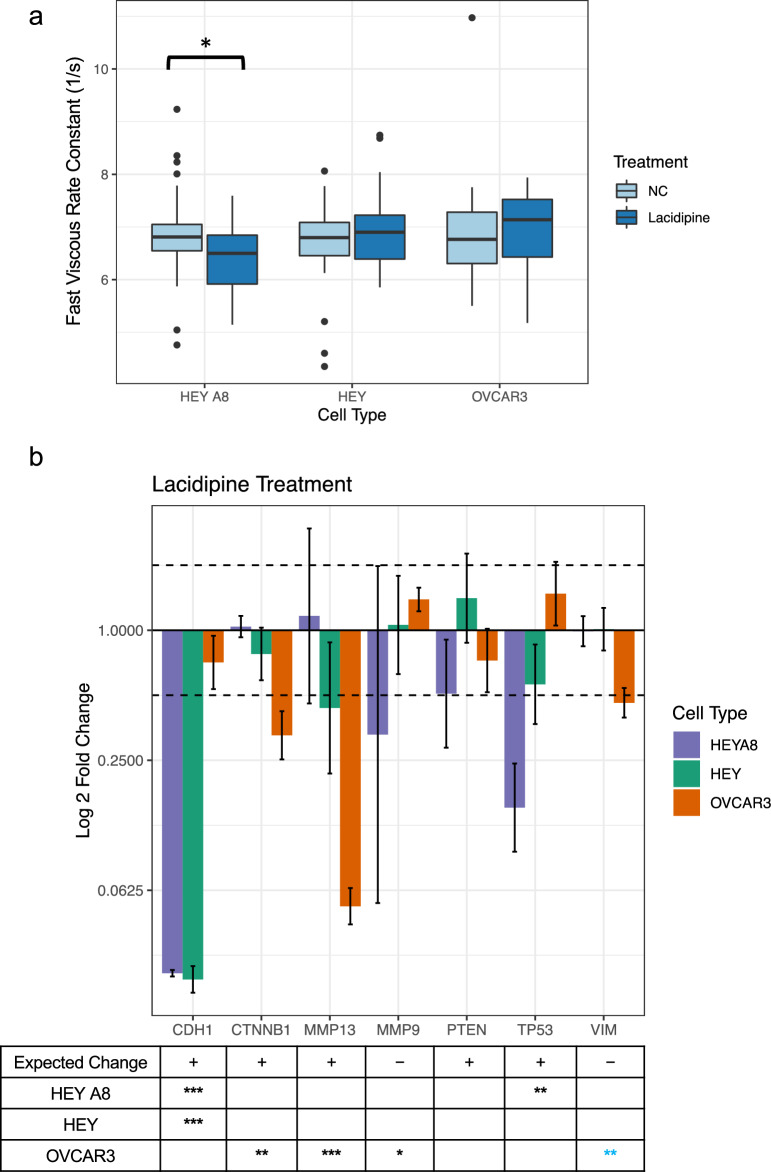
Lacidipine does not increase λ_1_ as predicted **a**) Boxplots comparing the fast viscous rate constant, λ_1_, of cells treated with a vehicle negative control and lacidipine for 24 h. n = 30 for each treatment condition. Lacidipine decreased λ_1_ for HEY A8 cells, but only resulted in a slight, non-significant increase of λ_1_ for HEY and OVCAR3 cells (Welch two sample t-test, *, p < 0.05). **b**) Cells were treated with lacidipine and RNA was extracted from cells 24 h post-treatment. RT-qPCR was used to detect the fold change differences of expression of predicted gene targets when normalized to housekeeping genes *GAPDH* and *RPL32* and expression levels of cells treated with a vehicle negative control. The two dotted lines indicate a twofold expression change threshold increase and decrease. Error bars represent the standard deviation of PCR data after error propagation. The table below the bar plot indicates the expected change in gene expression as predicted by the network analysis with the number of stars representing significant p-values for changes in gene expression between the negative control and treatment group after housekeeping gene normalization (Welch’s two sample t-test, *, p < 0.05, **, p < 0.01, ***, p < 0.001). Blue stars indicate genes that significantly changed in expression that align with the predicted change.

The network analysis predicted the small molecule chemical drug lacidipine would result in an increase of fast viscous rate constant λ_1_. We treated HEY A8, HEY, and OVCAR3 cells with 10 µM of lacidipine for 24 h followed by AFM measurement of the cell viscous properties. While we did see a slight, non-significant increase in λ_1_ in the HEY and OVCAR3 cell lines as predicted, we also observed a significant decrease in λ_1_ in the HEY A8 cell line, contrary to the predicted effect (Welch’s two sample t-test, HEY A8 – p = 0.022, HEY – p = 0.18, OVCAR3 – p = 0.92) (Fig. 2a). Further misalignment between the predicted cell behavior and the experimental validation was observed as several genes of interest were not affected as predicted by the IPA pathway (Supplementary Fig. 2d, Fig. 2b). One gene that was correctly aligned between the predicted behavior and measured expression after lacidipine treatment was a greater than twofold decrease in *VIM* expression in the epithelial OVCAR3 cell line. Similarly to the *AKT2* inhibition example above, the cell’s fast viscous response is related to a cell’s epithelial and mesenchymal qualities with a decrease in vimentin being associated with an increase of the fast viscous rate constant.

**Fig. 3:**
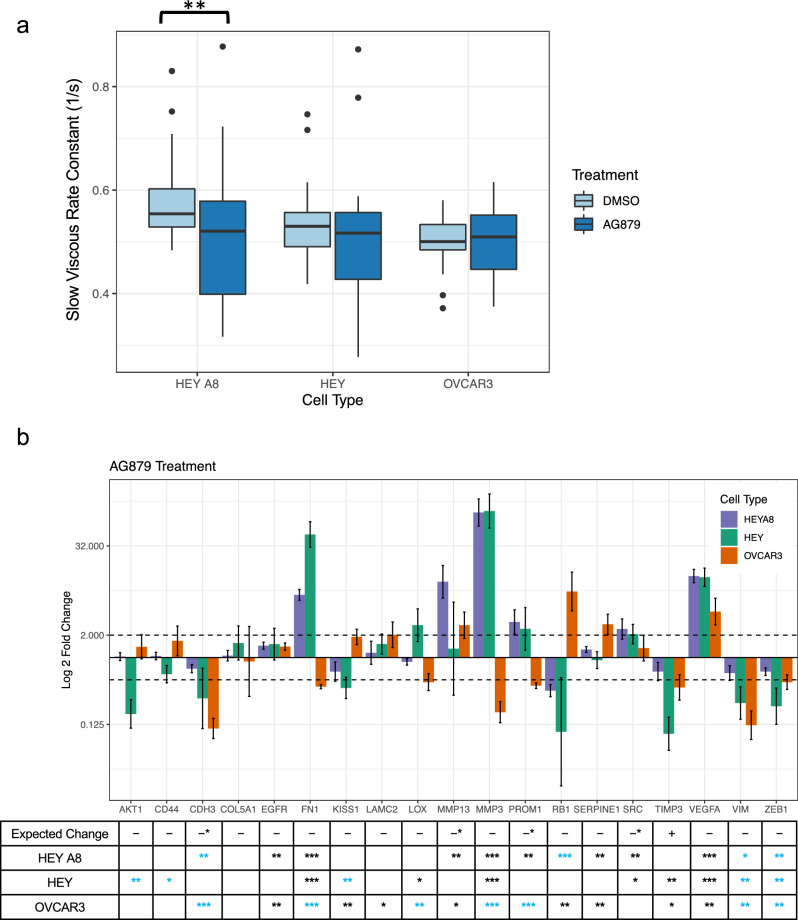
AG879 decreases λ_2_ as predicted for the HEY A8 cell line **a**) Boxplots comparing the slow viscous rate constant, λ_2_, of cells treated with small molecule chemical drug AG879. n = 40 for each treatment condition. AG879 resulted in a significant decrease in λ_2_ as predicted for the HEY A8 cell line (Welch two sample t-test, **, p < 0.01). There was a non-significant decrease in λ_2_ observed in the HEY cells, and little change observed in the OVCAR3 cell line. **b**) Cells were treated with AG879 and RNA was extracted from cells 24 hours post-transfection. RT-qPCR was used to detect the fold change differences of expression of predicted gene targets when normalized to housekeeping genes *GAPDH* and *RPL32* and expression levels of cells treated with a vehicle negative control. The two dotted lines indicate a 2-fold expression change threshold increase and decrease. Error bars represent the standard deviation of PCR data after error propagation. The table below the bar plot indicates the expected change in gene expression as predicted by the network analysis with the number of stars representing significant p-values for changes in gene expression between the negative control and treatment group after housekeeping gene normalization (Welch’s two sample t-test, *, p < 0.05, **, p < 0.01, ***, p < 0.001). Blue stars indicate genes that significantly changed in expression that aligns with the predicted change Asterisks in the expected change row denote genes negatively correlated with λ_2_ that were predicted to be downregulated.

The network analysis predicted AG879 treatment is expected to result in a decrease of slow viscous rate constant λ_2_. We treated HEY A8, HEY, and OVCAR3 cells with 10 µM of the small molecule chemical drug AG879 for 24 h followed by AFM measurement of the cell viscous properties. We observed a significant decrease in λ_2_ as predicted in HEY A8 cells, while we also saw a slight λ_2_ decrease in HEY cells, but little change in mechanics for the OVCAR3 cells after treatment (Welch’s two sample t-test, HEY A8 – p = 0.0030, HEY – p = 0.14, OVCAR3 – p = 0.93) (Fig. 3a). To help explain why we observed viscous changes in the HEY A8 and HEY cell lines but not the OVCAR3 cells, the gene expression change results showed a multiple genes in the HEY A8 and HEY cells decrease in expression as predicted, while OVCAR3 cells had an increase in expression in those particular genes, such as tumor suppressor gene and negative cell cycle regulator *RB1* and tumor and metastasis suppressor *KISS1* (Supplementary Fig. 3j, Fig. 3b). Interestingly, the network predicted a few genes that are negatively correlated with λ_2_, such as *SRC, PROM1,* and *MMP13,* would be downregulated by AG879, the predicted λ_2_ inhibitor. We would have expected these downregulated negatively correlated genes would work against the expected effect of decreasing λ_2_. However, when we experimentally tested the expression of these genes, we saw their expression increase for the HEY A8 and HEY cells, which would support the observed decrease in λ_2_. While the molecular expression only partially aligns with the network prediction, a predicted lowering in λ_2_ was observed for the mesenchymal cell types.

**Fig. 4:**
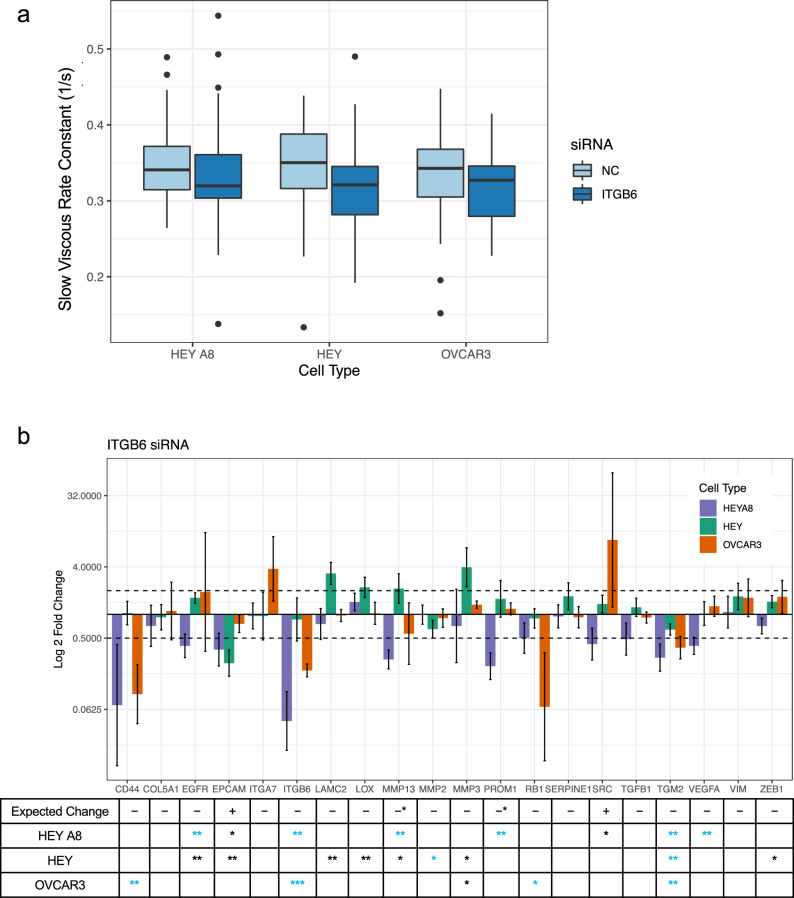
*ITGB6* knockdown leads to slight, but not significant, decrease in λ_2_ as predicted. **a**) Boxplots comparing the slow viscous rate constant, λ_2_, of cells transfected with siRNA against *ITGB6* for 48 hours. n = 40 for each treatment condition. *ITGB6* knockdown resulted in a slight, but not significant decrease in λ_2_ as predicted (Welch two sample t-test, p > 0.05). **b**) Cells were transfected with siRNA against *ITGB6*, and RNA was extracted from cells 48 hours post-transfection. RT-qPCR was used to detect the fold change differences of expression of predicted gene targets when normalized to housekeeping genes *GAPDH* and *RPL32* and expression levels of cells treated with a vehicle negative control. The two dotted lines indicate a 2-fold expression change threshold increase and decrease. Error bars represent the standard deviation of PCR data after error propagation. The table below the bar plot indicates the expected change in gene expression as predicted by the network analysis with the number of stars representing significant p-values for changes in gene expression between the negative control and treatment group after housekeeping gene normalization (Welch’s two sample t-test, *, p < 0.05, **, p < 0.01, ***, p < 0.001). Blue stars indicate genes that significantly changed in expression that align with the predicted change Asterisks in the expected change row denote genes negatively correlated with λ_2_ that were predicted to be downregulated.

The network analysis predicted a decrease in *ITGB6* expression that would result in the decrease of slow viscous rate constant λ_2_. We transfected HEY A8, HEY, and OVCAR3 cells with siRNA against *ITGB6* for 48 h followed by AFM measurement of the cell viscous properties. The knockdown of this integrin resulted in a decrease in λ_2_ as expected for all three cell lines, although the change was not statistically significant (Welch’s two sample t-test, HEYA8 – p = 0.41, HEY – p = 0.093, OVCAR3 – p = 0.18) (Fig. 4a). While we did see alignment of fold-expression changes for some genes of interest in some cell lines (such as down-regulation of *ITGB6, CD44, TGM2,* and *RB1*) (Supplementary Fig. 3d), there were also genes that significantly changed in a manner opposite to the predicted effect of *ITGB6*, including *EPCAM* down-regulation and up-regulation of *LAMC2, LOX,* and *MMP3* (Fig. 4b). Of note, *TGM2* was significantly downregulated in all three cells lines suggesting this crosslinking enzyme could be important for cell viscous relaxation.

### Network analysis can generate hypotheses to test putative targets that affect cell stiffness

In addition to exploring viscosity-related regulators, we applied a network analysis of the genes significantly correlated with cell stiffness to identify potential upstream targets that affect the expression of the correlated genes. The analysis resulted in 37 control nodes predicted to significantly affect cell mechanics, 11 of which we chose to represent because of their status as transcription factors, miRNAs, and other regulatory genes (Table 4 and Supplementary Fig. 4). Regulators with positive activation z-scores are associated with the increase of expression of genes positively correlated with stiffness or the decrease of expression of genes negatively correlated with stiffness; regulators with negative activation z-scores are expected to produce the opposite effect on genes of interest. To investigate whether the predicted regulators could affect cell stiffness as expected, we chose two regulators to test: *miR183*, predicted to decrease cell stiffness, and *CD82*, predicted to increase cell stiffness (Table 3). These regulators were selected because they would allow us to test both activators and inhibitors of cell stiffness that could be easily perturbed through treatment with a small molecule drug or through microRNA interference that was predicted to influence many genes correlated with the cell stiffness, potentially allowing these observed effects to be more clinically relevant. Additionally, these two regulators were predicted to perturb genes that were uniquely correlated with cell stiffness (such as *ACTA2, ACTB, AKT1, CD82, GSN, MYC, SNAI2,* and *TGFB1*), potentially allowing for the discovery of regulators that are specific to each mechanical parameter of interest.

**Table 4:** Network analysis reveals control nodes that could control cell stiffness.

Control node regulators	Molecule type	Activation z-score	Network bias-corrected p-value
RECK	Protease inhibitor	2.828	0.0006
MRTFA	Transcription regulator	2.500	0.0086
AR	Transcription regulator	2.111	0.0010
**CD82**	Metastasis suppressor	2.065	0.0009
SPEN	Transcription regulator	2.065	0.0074
miR520c	microRNA	2.000	0.0040
**miR183**	microRNA	−2.000	0.0014
EGFR	Kinase	−2.000	0.0005
ETV5	Transcription regulator	−2.333	0.0002
YAP1	Transcription regulator	−2.449	0.0041
TWIST2	Transcription regulator	−2.449	0.0009

Network analysis of genes correlated with stiffness revealed 37 significant potential control nodes, 11 of which are listed here. Bolded potential regulators were experimentally tested because of the accessibility of modification methods.

**Fig. 5:**
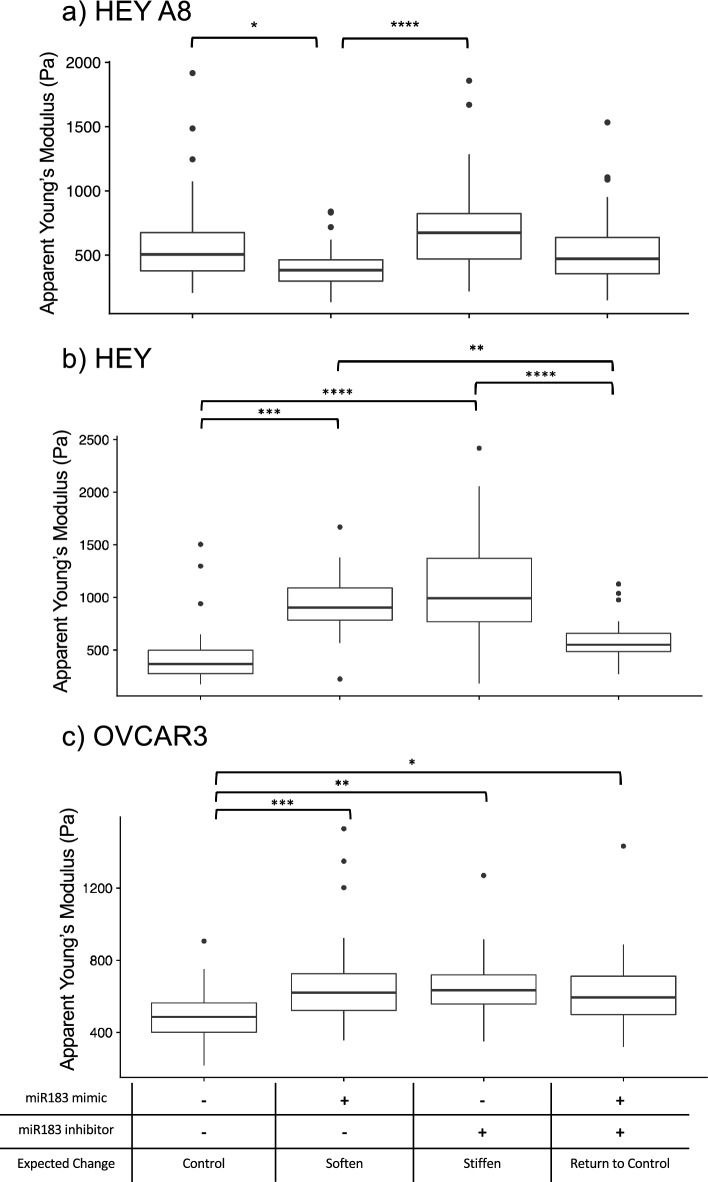
Changes in miR-183 expression result in cell stiffness changes in cell type specific manner. Boxplots comparing the apparent Young’s modulus of **a**) HEY A8, **b**) HEY, and **c**) OVCAR3 cells treated with combinations of negative control miRNC mimic, negative control miRNC inhibitor, *miR-183 *mimic and *miR-183 *inhibitor for 72 hours. N = 40 for each treatment condition. In HEY A8 cells, *miR-183 *mimic decreased cell stiffness while the *miR-183* inhibitor increased cell stiffness as expected. *miR-183* mimic and inhibitor transfection resulted in different responses in HEY and OVCAR3 cell lines (post-hoc Tukey HSD, *, p < 0.05, **, p < 0.01, ***, p < 0.001, ****, p < 0.0001).

To test the effect of modulation of *mir-183* on cell mechanics, we transfected HEY A8 cells with combinations of a mimic and an miRNA inhibitor. *mir-183 is* a control node with a negative activation z-score predicted to lead to a decrease in cell stiffness. HEY A8 cells transfected with the mature *miR-183* mimic for 72 h had a large increase of *miR-183* (41,866-fold change compared to cells transfected with negative control mimics and inhibitors, Supplementary Table 1) and were significantly softer than cells transfected with the negative controls as predicted (p < 0.05, post-hoc Tukey HSD) (Fig. 5a). Cells transfected with the *miR-183* inhibitor for 72 h demonstrated a 1.3-fold decrease in *miR-183* levels compared to cells transfected with both negative controls (Supplementary Table 1) and were significantly stiffer than the *miR-183* mimic transfected cells as expected (p < 0.0001, post-hoc Tukey HSD). When we transfected cells with both a *miR-183* mimic and *miR-183* inhibitor, cell stiffness remained unchanged (Fig. 5a). Interestingly, *miR-183* levels for the cells treated with both the mimic and inhibitor for *miR-183* were reduced as compared to cells treated with the *miR-183* mimic alone, but they were still highly elevated compared to cells that were transfected with both negative controls for the mimic and inhibitor (Supplementary Table 1). Additionally, when this same experiment was repeated with the HEY and OVCAR3 cell lines, we observed the inhibitor increased cell stiffness for the HEY and OVCAR3 cells as expected, though the mimic did not decrease cell stiffness in these cell lines (Figs. 5b and 5c).

**Fig. 6:**
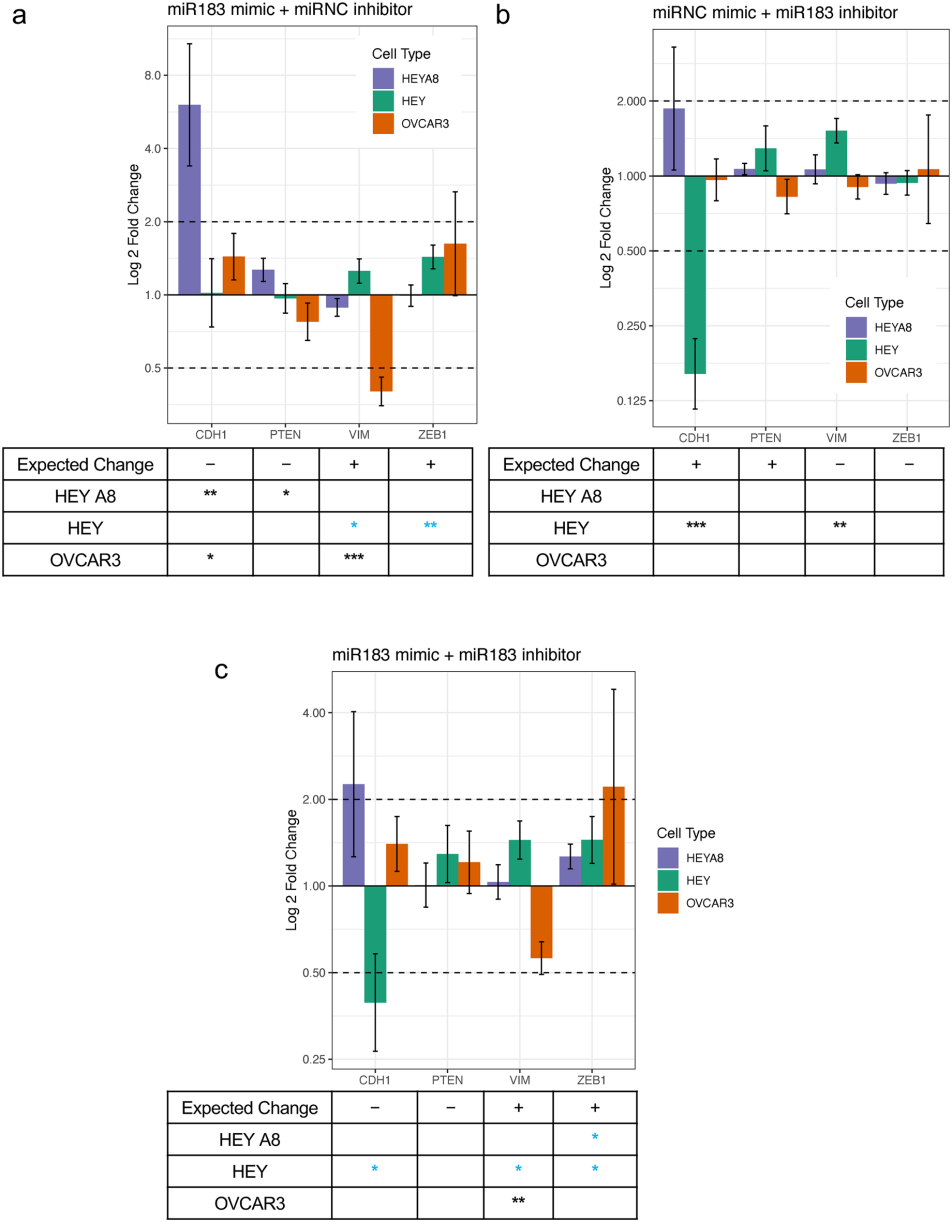
Gene expression did not change as expected with *MIR183* overexpression and inhibition. Cells were transfected with **a**) *MIR183 *mimic and a negative control miRNA inhibitor, **b**) *MIR183 *inhibitor and a negative control miRNA mimic, or **c**) both *MIR183 *mimic and inhibitor and RT-qPCR was used to detect the fold change differences of expression of predicted gene targets when normalized to housekeeping genes *GAPDH* and *RPL32* and expression levels of cells treated with both mimic and inhibitor negative controls. The two dotted lines indicate a 2-fold expression change threshold increase and decrease. Error bars represent the standard deviation of PCR data after error propagation. The table below the bar plot indicates the expected change in gene expression as predicted by the network analysis with the number of stars representing significant p-values for changes in gene expression between the negative control and treatment group after housekeeping gene normalization (Welch’s two sample t-test, *, p < 0.05, **, p < 0.01, ***, p < 0.001). Blue stars indicate genes that significantly changed in expression that aligns with the predicted change.

Although we did observe the expected stiffness changes in the HEY A8 cell line after transfection with the *miR-183* mimics and inhibitors, the expression of the downstream genes associated with this microRNA (*CDH1, PTEN, VIM,* and *ZEB1*) were not regulated in accordance with the predictions from the network analysis (Supplementary Fig. 4 g and Fig. 6). For example, we observed a greater than twofold increase of *CDH1* expression in the HEY A8 cells when the network analysis predicted *miR-183* to be associated with a decrease of *CDH1.* Also, we observed a greater than twofold decrease of *VIM* expression in OVCAR3 cells when it was expected to increase. Therefore we confirmed the mechanical regulation hypothesis of *miR-183* as a cell softener, but only in the HEY A8 cell line and not through the hypothesized set of gene regulation.

**Fig. 7:**
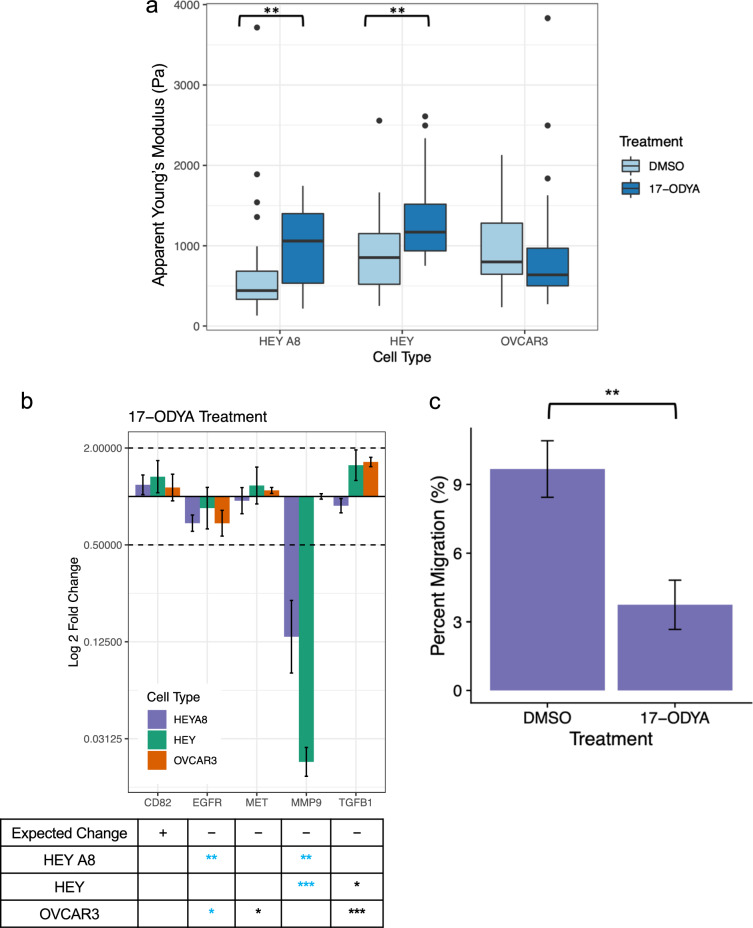
Cell stiffening 17-ODYA treatment results in gene expression changes that are slight, but consistent with predictions and reduces cell migration. **a**) Boxplots comparing the apparent Young’s modulus of cells treated with a negative control DMSO vehicle and of those treated with small molecule inhibitor and CD82 up-regulator 17-ODYA. n = 40 for each treatment condition. 17-ODYA increased cell stiffness (Welch two sample t-test, **, p < 0.01) for HEY A8 and HEY cell lines, but not OVCAR3 cell line. **b**) Cells were treated with 10 µM 17-ODYA for 24 hours and RT-qPCR was used to detect the fold change differences of expression of predicted gene targets when normalized to housekeeping genes *GAPDH* and *RPL32* and expression levels of cells treated with DMSO vehicle control. The two dotted lines indicate a 2-fold expression change threshold increase and decrease. Error bars represent the standard deviation of PCR data after error propagation. The table below the bar plot indicates the expected change in gene expression as predicted by the network analysis with the number of stars representing significant p-values for changes in gene expression between the negative control and treatment group after housekeeping gene normalization (Welch’s two sample t-test, *, p < 0.05, **, p < 0.01, ***, p < 0.001). Blue stars indicate genes that significantly changed in expression that aligns with the predicted change **c**) Transwell migration assay data demonstrate the decreased migratory ability of the HEY A8 cells treated with 17-ODYA compared to the HEY A8 cells treated with a negative control DMSO vehicle (Welch two sample t-test, **, p < 0.01). Cells that migrated through the transwell were fluorescently quantified and normalized using a standard curve.

To test another control node, we treated HEY A8, HEY, and OVCAR3 cells with the small molecule 17-octadecynoic acid (17-ODYA), an inhibitor of *CYP2J2*, to overexpress the potential control node regulator *CD82* with a positive activation z-score that was hypothesized to result in cell stiffening. After 24 h of treatment with 17-ODYA, all three cell lines demonstrated an increase of *CD82* expression (Fig. 7b) and both the HEY A8 and HEY cell lines treated with 17-ODYA were significantly stiffer than cells treated with a DMSO vehicle control (HEY A8 – p = 0.0066, HEY – p = 0.0056, Welch two sample t-test) (Fig. 7a). However, the treated OVCAR3 cells did not significantly change in mechanical properties. Investigating the downstream gene targets of 17-ODYA can help explain some of this cell type specificity (Supplementary Fig. 4d). For the HEY and HEY A8 cell types, the downstream genes were regulated in the manner hypothesized by the network analysis with an increase of *CD82* expression and decreased expression of *EGFR, MET, MMP9,* and *TGFB* (Fig. 7b). However, for OVCAR3 cells, we did not observe a reduction in *MMP9* expression, supporting an explanation for the different mechanical response of OVCAR3 cells to 17-ODYA treatment. Finally, we also investigated the functional effect of 17-ODYA on cell migration. After treatment with 17-ODYA, HEY A8 cells were significantly less migratory than cells treated with a DMSO vehicle control (p = 0.0033, Welch two sample t-test) (Fig. 7c). This result supports the hypothesis that there is a link between cell softening and an increasingly migratory phenotype which may also be connected to a cell’s ability to remodel its extracellular environment.

## Discussion

This study focuses on the novel exploration of the relationship between the viscoelasticity of cells and the underlying molecular mechanisms that affect cell mechanics. From a network analysis, we identified several putative gene and small molecule regulators that affect multiple genes significantly correlated with cell viscous properties and with cell stiffness. The viscoelastic properties of cells are determined by several cellular components, such as the cell membrane, cytoskeleton and viscosity of their cytoplasm. Two examples of this include the active contraction of the cytoskeleton resulting in intracellular poroelasticity and differences in cytoskeletal fluidity based on differences in non-covalent bonds holding together the various filaments^17–19^. The fast and slow viscous rate constants capture relaxation responses of different cellular components, with the fast viscous rate constant thought to be associated with the relaxation of the cell membrane while the slow viscous rate constant is in line with the relaxation time scale of cytoskeletal rearrangement^20^. To explore potential control of these cellular mechanical properties, we focused our study on six predicted regulators – *ATK2*, Lacidipine, AG879, *ITGB6*, *miR183* and *CD82*.

These six predicted regulators represent both activators and inhibitors of our three mechanical properties of interest, the fast viscous rate constant, the slow viscous rate constant, and cell stiffness. This combination of gene regulator targets and small molecule regulators was chosen because they could be simply perturbed by treatment with a small molecule drug, through RNA interference with a commercially available, tested siRNA or through microRNA interference. We surmised that simple modulators of the target pathways would allow for a simpler translation to future preclinical and clinical testing through multiple types of potential treatments. Furthermore, these six regulators were predicted to perturb genes that were specifically correlated with each mechanical parameter (Supplementary Fig. 1). We reasoned that the discovery of regulators that were more specific to each mechanical property of interest would further support future translation. Interestingly, there are other related predicted regulators that would also be worthwhile to explore in future work, such as *YAP1, WWTR1* (*TAZ),* and *TEAD2*. *YAP* and *TAZ* are mechanosensitive transcriptional coactivators in the Hippo signaling pathway that interact with the *TEAD2* transcription factor, which binds to DNA when activated to promote changes in gene expression. These genes have been closely linked to cancer cell mechanoadaptability during the metastatic cascade with *YAP1* inhibition leading to reduced cell stiffness in response to fluid shear stress and reduced migration and extravasation^21^. However, we decided to not choose *YAP1, TAZ,* or *TEAD2,* as node regulators to perturb in our current study because of their predicted effect on both cell stiffness and cell viscous properties (see Table 2 and Table 4).

Our first regulator node perturbation, *AKT2* inhibition, showed great promise for increasing the cell viscous relaxation rate as measured by the λ_1_ parameter in multiple cell lines. Evaluating whether these viscosity changes have functional relevance in cell migration could be further explored. *AKT2* inhibition has been linked to decreased mesenchymal stem cell, breast cancer, and lung cancer cell migration, therefore we may anticipate that increased viscous relaxation rate would correlate with decreased migratory capacity^22–24^. Some differences were observed in gene expression changes across cell types which appeared to be related to their epithelial-mesenchymal status. *AKT2* knockdown has been associated with the inhibition of the epithelial to mesenchymal transition (EMT)^25^. The mesenchymal cells lines HEY and HEYA8 started with lower level of E-Cadherin prior to *AKT2* inhibition, so while the *CDH1* expression decreases for these cells, it may be that there are additional gene pathways at play that also contribute to the specific changes to each cell line’s EMT status and viscous properties.

Our next target node was the small molecule drug, lacidipine, a voltage-gated calcium channel blocker, which is commonly used as an anti-hypertensive agent, but has recently been studied as a novel therapeutic targeting ovarian cancer stem cells^26,27^. Lacidipine reduced the stemness and chemoresistance of ovarian cancer cells through AKT-ERK signalling and the related EMT changes described above. After lacidipine treatment, there was a twofold decrease of vimentin in the epithelial OVCAR3 cell line that aligned with the predicted gene network behavior. Again, it appears that with *AKT2* inhibition, through BAY1125976 or the predicted lacidipine drug, the cell’s fast viscous response is related to a cell’s epithelial status with a more epithelial and less mesenchymal gene expression associated with an increase of the fast viscous rate constant. This aligns with a recent observation that more epithelial colorectal cells had cell membranes with lower microviscosity (corresponding to a higher fast viscous rate constant) that was associated with a low intrinsic motility^28^.

To affect the slow viscous rate constant instead of the fast viscous rate constant, we used the predicted small molecule drug AG879, a tyrphostin-kinase inhibitor specific for *ERBB2* and a *VEGF* receptor^29^. A reduction of the slow viscous rate constant was observed in the mesenchymal HEY A8 and HEY cells as expected, while no change was observed in the epithelial OVCAR3 cells. The mesenchymal cells demonstrated a decrease in expression of tumor suppressor and negative cell cycle regulator *RB1* and tumor and metastasis suppressor *KISS1* while OVCAR had an increase in expression of these two genes. Downregulation of these genes has been associated with EMT in triple negative breast cancer and ovarian cancer and increased tumor growth and metastasis in non-small cell lung cancer and hepatocarcinoma^30–33^. Therefore, for mesenchymal cells, we would expect that the reduction of the slow viscous rate constant after AG879 treatment that reduces *RB1* and *KISS1* expression may lead to increased cancer cell migration and metastasis.

The final viscous node regulator we perturbed was *ITGB6,* the integrin subunit beta-6 that associates with integrin alpha-v to mediate cell–cell and cell–matrix adhesion events. While we did see a slight reduction in cell viscous properties after knockdown of *ITGB6,* we may see greater effect on mechanics and possibly also migratory phenotypes if we targeted both components of the integrin heterodimer. One study found that the heterodimer of *ITGAV* and *ITGB6* are required for neoplastic invasion of epidermal squamous cell carcinoma^34^. After *ITGB6* knockdown, the gene *TGM2* was significantly downregulated in all three cell types, In a mouse model of osteosarcoma, both *ITGB6* and *TGM2* were found to be similarly overexpressed in distal metastatic sites compared to primary lesions, implying further correlation and connection between these proteins of interest^35^. Further investigation is warranted to understand how these proteins involved so heavily in interactions with the cells’ external environment also are correlated with and affect the cells’ internal viscoelastic properties.

In addition to exploring the molecular mechanisms related to cell viscosity, we found that the overexpression of *miR-183* would lead to cell softening in the HEY A8 cell line. *miR-183* has been found to be overexpressed in most breast cancers and has been categorized as an oncogene in multiple cancer types for its contribution to increased cell proliferation and migration^36–38^. Additionally, in ovarian cancer, downregulation of the microRNA has been shown to inhibit cell proliferation, migration, and invasion^39^. However, the role of *miR-183* in metastasis is complex, with some groups identifying the microRNA as a tumor suppressor in osteosarcoma and lung cancer with observation that overexpression of the miR resulted in transition to a more epithelial and less metastatic phenotype^40,41^. Interestingly, the change in cell stiffness seems not to be mediated through the genes predicted by the network analysis. We anticipate that the biological effect of miRNAs can be cell line dependent since the Ingenuity Pathway Analysis network is based upon the accumulated data from many cell types. Moreover, the expression of genes is time dependent; while we chose each analysis timepoint as an expectation of expression dynamics, it is possible the relevant changes were missed by our measurements. Finally, the action of miRNA can be both dose dependent and non-monotonic, therefore alterations in dosing may change the observations^42^. Our work demonstrating *miR-183*’s role in cell softening in the metastatic, mesenchymal HEY A8 cell line supports the connection between a soft mechanotype, expression of genes in the EMT pathway and a more metastatic phenotype.

Additionally, we identified 17-ODYA as a cell stiffening small molecule inhibitor through its minor up-regulation of *CD82* for the mesenchymal cell lines HEY and HEY A8. The metastasis suppressor gene *CD82* has been implicated in several cancers to inhibit cell migration through restricting cell protrusion and retraction^43,44^. Both *CD82* and *MMP9* expression increase with the treatment of the epoxygenase inhibitor 17-ODYA which has also been associated with inhibition of cell migration and invasion^45^. We do note the lack of mechanical effect of 17-ODYA treatment on the epithelial OVCAR3 cell line. One explanation may involve the cell state and nascent gene expression levels prior to treatment. For example, 17-ODYA resulted in large increases of *CDH1* in the HEY and HEY A8 cell lines. However, E-Cadherin is already highly expressed in the epithelial OVCAR3 cell line so 17-ODYA perturbation would be expected to produce less of a practical effect on this cell line. The ability of 17-ODYA to stiffen the mesenchymal cell lines and reduce their migratory phenotype suggests this inhibitor represents a potential therapeutic agent for metastasis prevention through a genomechanical pathway. The effects of each potential regulator on both cel mechanics and gene expression changes are summarized in Table 5.

**Table 5:** Summary of hypothesis testing of control node regulation of mechanics and gene expression.

		*AKT2*	Lacidipine	AG879	*ITGB6*	*miR183*	*CD82*
Cell mechanics	HEY A8	Aligned: Significant	Misaligned: Significant	Aligned: Significant	Aligned: Not significant	Aligned: Significant	Aligned: Significant
	HEY	Aligned: Not significant	Aligned: Not significant	Aligned: Not significant	Aligned: Not significant	Misaligned: Significant	Aligned: Significant
	OVCAR3	Aligned: Significant	Aligned: Not significant	No change	Aligned: Not significant	Misaligned: Significant	Misaligned: Not significant
Gene changes	HEY A8	Misaligned	Misaligned	Mixed alignment	Mixed alignment	Misaligned	Aligned
	HEY	Mixed alignment	Misaligned	Mixed alignment	Mixed alignment	Aligned	Mixed alignment
	OVCAR3	Aligned	Mixed alignment	Mixed alignment	Aligned	Misaligned	Mixed alignment

Table summarizing the effect of each potential node regulator on the mechanical properties and gene expression changes as predicted by the network analyses. Alignment with predicted gene expression changes based on significant changes (p < 0.05, Welch’s two sample t-test).

Overall, this study used a systems biology network approach to investigate biomechanical mechanisms by testing several hypotheses linking cell mechanical properties to the expression of genes that can be manipulated with pharmacological agents or gene therapies. Many of these genes also play a role in ovarian cancer cell metastasis. We identified a number of cell line specific regulation points that have an effect on cell viscous properties (e.g. *AKT2, ITGB6*, and AG879) and on cell stiffness (e.g. *miR-183* and 17-ODYA). We also validated the impact on cancer cell migration by molecular, mechanical, and functional assays. Further study into why these regulators have different effects in different cell lines remains and specifically, how improved models for the network analysis may result in more accurate predicted relationships between mechanical and gene expression measurements.

## Methods

### Single cell genomechanics dataset

A data set was generated from a single cell genomechanical method^15^ and we identified 12 genes significantly correlated with fast viscous rate constant parameter λ_1_, 32 genes correlated with slow viscous rate constant parameter λ_2_, and 39 genes correlated with cell stiffness (p < 0.05, Spearman’s correlation test) (Supplementary Fig. 1). The data sets were acquired from single-cell atomic force microscopy (AFM) measurements on 273 ovarian cancer cells from three cell types (91 HEY A8 cells, 95 HEY cells, 87 OVCAR3 cells). We extracted cell viscous properties and stiffness by applying standard linear solid viscoelastic and Hertzian contact mechanics models, described further below. Each single cell was isolated using micropipette aspiration for downstream analysis of 85 genes of interest related to cytoskeletal proteins, cancer pathways, cell mechanics, and metastatic processes. After normalizing each cell’s expression to its housekeeping gene, we examined the correlative relationships between the mechanical properties and the genes probed at the single-cell level using Spearman’s pairwise correlation.

### Gene network analysis

We applied a Causal Network Analysis^46^ from the Ingenuity Pathway Analysis package using the genes significantly correlated with both cell viscous properties and cell stiffness to identify potential upstream regulators that can regulate the expression of several of these genes. The network analysis was applied to genes correlated with the fast viscous rate constant, the slow viscous rate constant and cell stiffness, and predicted causal networks were selected as significant with network-corrected p-value < 0.01 (right-tailed Fisher’s exact test) and absolute Z-scores ≥ 2. The overlap p-value can be interpreted as a probability that a given control node is associated with downstream genes correlated with each cell mechanical property by chance. In addition, network bias-corrected p-values can be calculated, which correct overlap p-values for bias introduced by downstream genes with“hub character”, i.e., downstream genes regulated by many control nodes^46^. For control node regulators, the network bias-corrected p-values imply very low probability of associations of each of these regulators with mechanics-regulated genes only by chance.

### Ovarian cell culture

The ovarian cancer cell lines OVCAR3, HEY, and its derivative cell line HEY A8 were cultured in RPMI-1640 media (Sigma-Aldrich) supplemented with 10% fetal bovine serum (Atlanta Biologicals) and 1% penicillin–streptomycin (Sigma-Aldrich). Cells were cultured at 37℃ at 5% CO_2_. All three cell lines were provided by Dr. John McDonald (Georgia Institute of Technology, Atlanta, GA). The HEY and HEY A8 cell lines were provided originally from Dr. G Mills (MD Anderson Cancer Center, Houston, TX) and the OVCAR3 line was procured originally from the Developmental Therapeutic Program (DTP of the National Cancer Institute (NCI)) (Bethesda, MD). For AFM experiments, approximately 50,000 cells were seeded in a glass bottomed FluoroDish (World Precision Instruments) for 24-h treatment protocols. For RNA extraction, approximately 250,000 cells were seeded in a T25 flask (VWR) for 24-h treatment protocols.

### Control node perturbations

We tested the effect of modulation of multiple potential control nodes of cell mechanics and gene expression, including: genes (*AKT2, ITGB6,* and *CD82)*, small molecule inhibitors (lacidipine and AG879), and precursor microRNAs (mir-183)*.*

*AKT2* down-regulation was induced by treatment of cells with 10 μM BAY1125976 (MedChemExpress) reconstituted in DMSO. An equivalent amount of DMSO was used as a vehicle control. After 24 h of treatment, as recommended, cell viscosity was measured with AFM as described below.

Lacidipine, a dihydropyridine calcium antagonist, was used as a regulator of genes correlated with λ_1_. Cells were treated with 10 μM lacidipine (Sigma Aldrich) reconstituted in DMSO. An equivalent amount of DMSO was used as a vehicle control. After 24 h of treatment, as recommended, cell viscosity was measured with AFM as described below.

AG879, a tyrosine kinase inhibitor, was used as a regulator of genes correlated with λ_2_. Cells were treated with 10 μM Tyrphostin AG 879 (Sigma Aldrich) reconstituted in DMSO. An equivalent amount of DMSO was used as a vehicle control. After 24 h of treatment, as recommended by the manufacturer, cell viscosity was measured with AFM as described below.

*ITGB6* knockdown was induced by treating cells via transfection with *ITGB6* siRNA Silencer assay 144,658 or Silencer Select Negative Control No. 1 siRNA (ThermoFisher). We determined optimal transfection conditions to be 1.7 μL of Lipofectamine RNAiMAX Transfection Reagent (Life Technologies) and 33 nmol of siRNA per 1 mL of media as well as the optimal incubation time post-transfection. Cell viscosity was measured 48 h post-transfection with AFM as described below.

Mature microRNA miR-183 was overexpressed in cells via transfection with mirVana miRNA mimic for hsa-miR-183-5p (ThermoFisher). Alternatively, cells were transfected with a negative control mimic miRNA (ThermoFisher). Additionally, we transfected cells with mirVana miRNA inhibitor for hsa-MIR183-5p or a negative control inhibitor miRNA at a ratio of 1:5 mimic to inhibitor. We determined optimal transfection conditions to be 2 μL of Lipofectamine RNAiMAX Transfection Reagent (Life Technologies) and 30 pmol of miRNA per 1 mL of media as well as the optimal incubation time post-transfection. After 6 h, the transfection media was exchanged for fresh media, and cell stiffness was measured 72 h post-transfection with atomic force microscopy, as described below.

*CD82* up-regulation was induced by treating cells with 50 μM 17-octadecynoic acid (17-ODYA) (Cayman Chemical) reconstituted in DMSO (Sigma Aldrich). An equivalent amount of DMSO was used as a vehicle control. 17-ODYA is an inhibitor of *CYP2J2,* an inhibitor of CD82 expression; therefore, 17-ODYA upregulates the expression of CD82^45^. After 24 h of treatment, cell stiffness was measured with AFM as described below.

### AFM and force curve analysis

To characterize the mechanical properties of each cell, we used force spectroscopy to obtain force-indentation curves with an atomic force microscope (Asylum Research) with an integrated optical microscope (Nikon) on a vibration isolation table. For improved global stiffness measurements of the cell, 5.24 μm spherical silica particles were attached to tipless silica nitride cantilevers (Bruker Probes) using a two-part epoxy. Each cantilever was calibrated to determine the deflection inverse optical lever sensitivity and spring constant (k is approximately 10–25 pN/nm). For measurements, the cantilever probe was visually aligned with the cell center and translated with a velocity of 2 μm/s to indent the cell with increasing compressive force until a force trigger of 5 nN was reached. This constant force trigger resulted in an indentation depth of approximately 2–6 µm with an average indentation depth of 2.5 µm, which is equivalent to approximately 25% of the average cell height. The cantilever was held in position for 10 s, dwelling towards the surface, to record the viscous relaxation of the cell before reversing the probe velocity.

We used custom code written in R (https://github.com/nstone8/Rasylum) relying on the Hertzian contact model, which describes non-adhesive elastic contact between two bodies, to calculate the cellular apparent Young’s modulus^47^. The contact point was estimated by the intersection of the flat, undeformed region of the force curve with a line fit to the force curve region where the cantilever was in contact with the cell. Next, we identified the true contact point by iteratively testing the points around the estimated contact point with the minimal residual difference between the measured force curve and a nonlinear fit described by the governing equation Hertz used to describe the contact between an elastic sphere and an elastic half space:

$$F=\frac{4}{3} {E}^{*}\sqrt{r{d}^{3}}$$

where F is the force measured by the AFM, E* is the calculated apparent Young’s modulus, r is the radius of the spherical tip, and d is the indentation measured by the z-position of the AFM.

We additionally used the Rasylum custom R package to fit the dwell region of the force curve to a biexponential decay curve that describes the relaxation of a standard linear solid model that is a parallel combination of an elastic element (represented by a spring) with two Maxwell viscoelastic elements (represented by a spring and dashpot in series) to identify the fast (λ_1_) and slow (λ_2_) viscous rate constants^48^. The equation we used to fit the viscous relaxation data was:

$$F\left(t\right)=C+\left({F}_{0}-C\right)[A\text{exp}\left(-\left(t-{t}_{0}\right)\left({\lambda }_{1}\right)\right)+(1-A)\text{exp}\left(-\left(t-{t}_{0}\right)\left({\lambda }_{2}\right)\right)]$$

where F is the force measured by the AFM, C is the fitted parameter equivalent to the asymptote of the exponential decay, F_0_ is the initial force before viscous relaxation, A is a fitted parameter, λ_1_ is the fast viscous time constant and λ_2_ is the slow viscous time constant. The larger the value of the rate constants, the faster the viscous relaxation. The residual sum of squares for each viscous fit was less than 5 × 10–^17^.

### RNA Extraction and RT-qPCR

To compare the gene expression of each cell line after perturbation of each control node, we used qPCR with SsoAdvanced Universal SYBR Green Supermix (BioRad). mRNA was isolated using Trizol™ Reagent (ThermoFisher) from HEY, HEY A8 and OVCAR3 cells grown in T-25 cell culture treated flasks (approximately 2 million cells per each flask). mRNA was reverse transcribed using the iScript cDNA synthesis kit (BioRad) that utilizes a blend of oligo dTs and random hexamer primers. An initial 30 s melt phase at 95℃ was followed by 40 cycles of a 10 s melt phase at 95℃ and a 30 s anneal and extend phase at 60℃ with a final melt curve analysis afterwards. All primers used for downstream gene analysis were previously validated and listed^15^. Amplification curves from the ABI StepOne Plus qPCR machine were analyzed using LinRegPCR (https://www.medischebiologie.nl/files/) to determine a C_t_ for each sample-assay pair. The ΔΔC_t_ method was used to compare the fold-change expression differences between each treatment and control group. In short, we took the difference of the C_t_ value of the geometric mean expression of housekeeping genes *GAPDH* and *RPL32* from the gene of interest’s C_t_ value. Next, we took the difference between the normalized values of the control and treatment groups. Finally, we calculated the relative fold gene expression values using the expression 2^-ΔΔCt^. Error propagation was used to determine the standard deviation of the relative fold gene expression values and a Welch’s two sample t-test was used to determine significant changes in gene expression between the negative control and treatment group expression values after normalization to housekeeping genes. miRNA expression was measured using RT-qPCR with TaqMan fluorescent probes (Life Technologies) with UNG activation and normalization to *RNU6B* (ThermoFisher catalog 4,427,975, assay ID 001,093) expression. miRNA was reverse-transcribed using the TaqMan MicroRNA Reverse Transcription Kit (Applied Biosystems catalog number 4366596) with the qPCR assay ID 002,269 primers for hsa-miR-183 (ThermoFisher catalog number 4427975) at 16℃ for 30 min, 42℃ for 30 min, and 85℃ for 5 min. qPCR was then completed with the TaqMan Universal Master Mix (with UNG) (ThermoFisher catalog 4,440,042). UNG activation was completed at 50℃ for 2 min, followed by enzyme activation at 95℃ for 10 min and ending with 40 cycles of denaturing at 95℃ for 15 s and annealing and extending at 60℃ for 60 s.

### Migration assay

To test the migratory properties of cells after 17-ODYA treatment, we used the Cultrex 24-well transwell migration assay (Trevigen), where 100,000 cells of each cell population were seeded in serum free media (RPMI-1640) in an 8-micron pore well insert, leading to a well filled with complete media (RPM1-1640 media supplemented with 10% FBS and 1% penicillin–streptomycin). After 8 h of incubation, the cells that migrated through the pores were detached, lysed and stained with Calcein-AM fluorescent dye. The amount of fluorescence per well was read with a microplate reader at 480 nm/520 nm (BioTek Synergy H4). A standard curve was performed to determine the relationship between fluorescence and number of cells.

## Supplementary Information

Supplementary Information.

## Data Availability

The original contributions presented in this study are included in the article and supplementary material. Further inquiries can be directed to the corresponding author.
