# Supplementary material for: Network analysis of gene expression reveals regulators of cell viscosity and mechanical phenotype
Source: Sci Rep. 2025 Sep 30;15:34008. doi: 10.1038/s41598-025-11698-0 (PMC12484610; doi:10.1038/s41598-025-11698-0)
Supplement: Supplementary file 1 — Supplementary Information. [file 41598_2025_11698_MOESM1_ESM.zip › FigureS1.pdf]

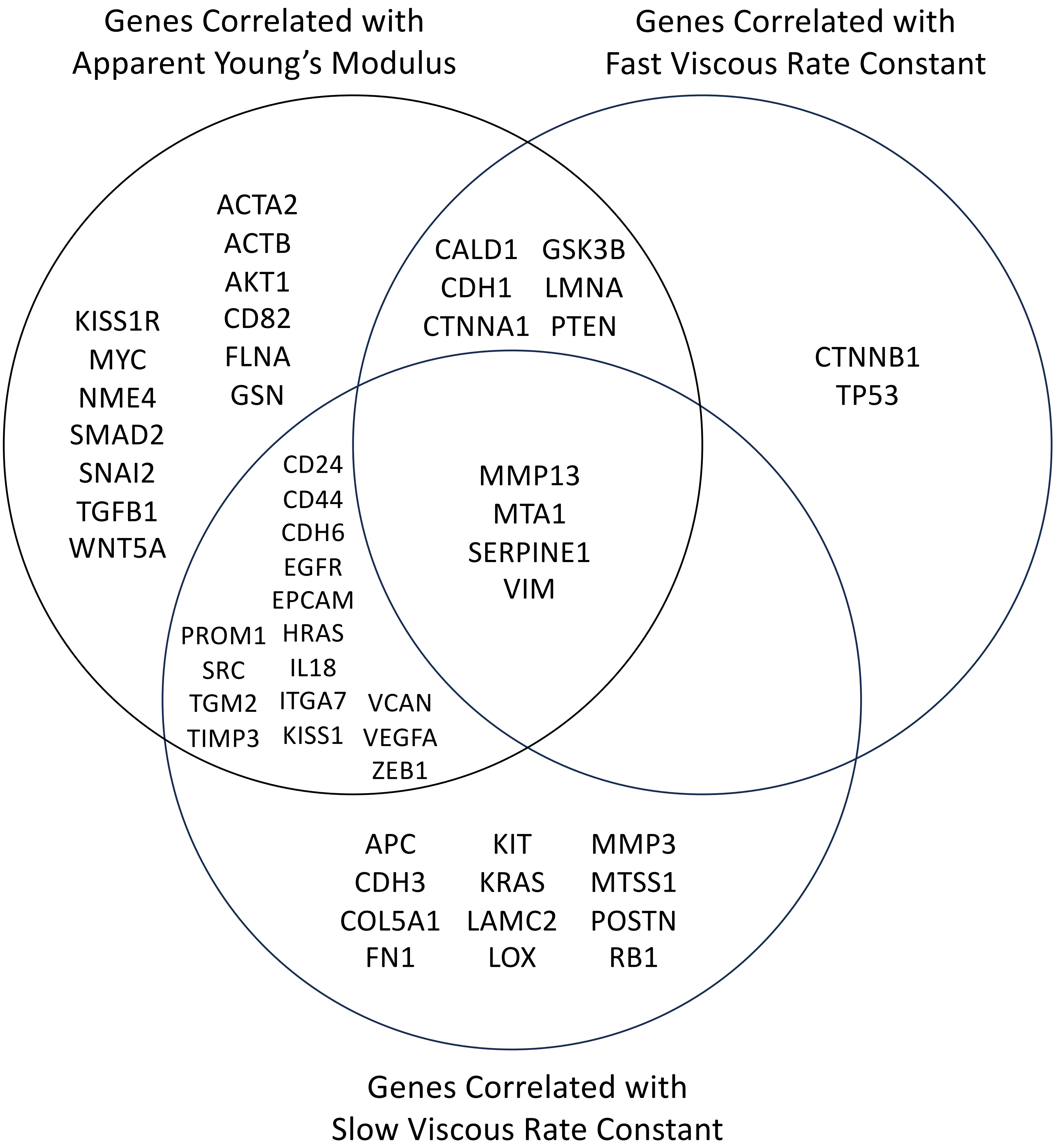

Supplementary Figure 1 - Venn Diagram of Genes Correlated with Cell Mechanical Properties -

In our previous work, Young et. al 2023, we determined a subset of genes significantly correlated with cell apparent Young's modulus, cell fast viscous rate constant, and cell slow viscous rate constant as determined by the method of single cell genomechanics which combines data collected from atomic force microscopy and multiplexed RT-qPCR.
