# Supplementary material for: Network analysis of gene expression reveals regulators of cell viscosity and mechanical phenotype
Source: Sci Rep. 2025 Sep 30;15:34008. doi: 10.1038/s41598-025-11698-0 (PMC12484610; doi:10.1038/s41598-025-11698-0)
Supplement: Supplementary file 1 — Supplementary Information. [file 41598_2025_11698_MOESM1_ESM.zip › FigureS2.pdf]

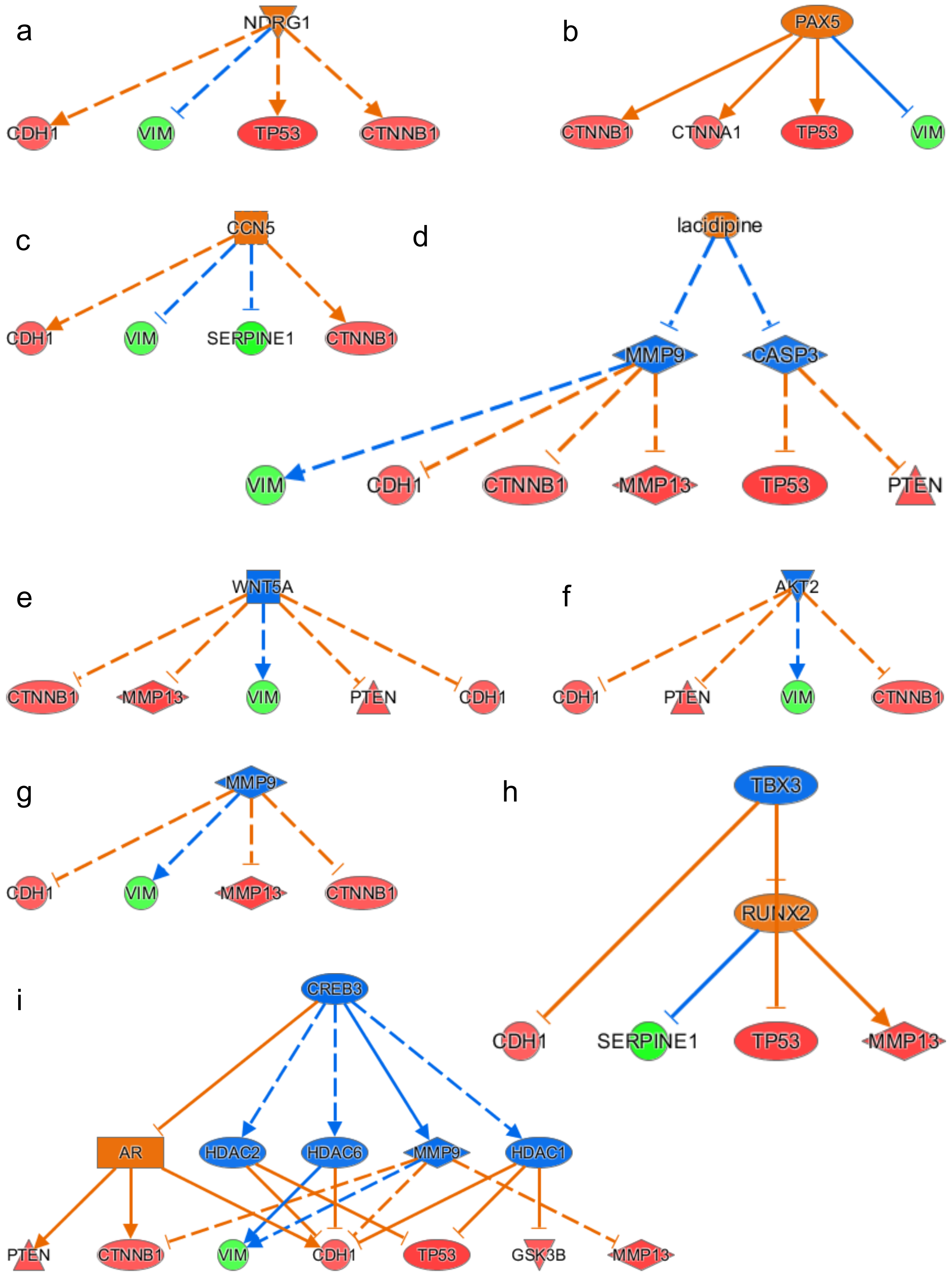

Supplementary Figure 2 - Network of genes predicted to be under the control of tau1 control nodes – a) *NDRG1*, b) *PAX5*, c) *CCN5,* d) lacidipine, e) *WNT5A*, f) *AKT2*, g) *MMP9,* h) *TBX3,* and i) *CREB3*. Blue nodes represent regulators predicted to increase tau1. Orange nodes represent regulators predicted to decrease tau1. Green nodes are genes negatively correlated with tau1 and red nodes are genes positively correlated with tau1. Dashed lines indicate indirect interactions while solid lines indicate direct interactions as demonstrated in previous literature.
