# Supplementary material for: Network analysis of gene expression reveals regulators of cell viscosity and mechanical phenotype
Source: Sci Rep. 2025 Sep 30;15:34008. doi: 10.1038/s41598-025-11698-0 (PMC12484610; doi:10.1038/s41598-025-11698-0)
Supplement: Supplementary file 1 — Supplementary Information. [file 41598_2025_11698_MOESM1_ESM.zip › FigureS3.pdf]

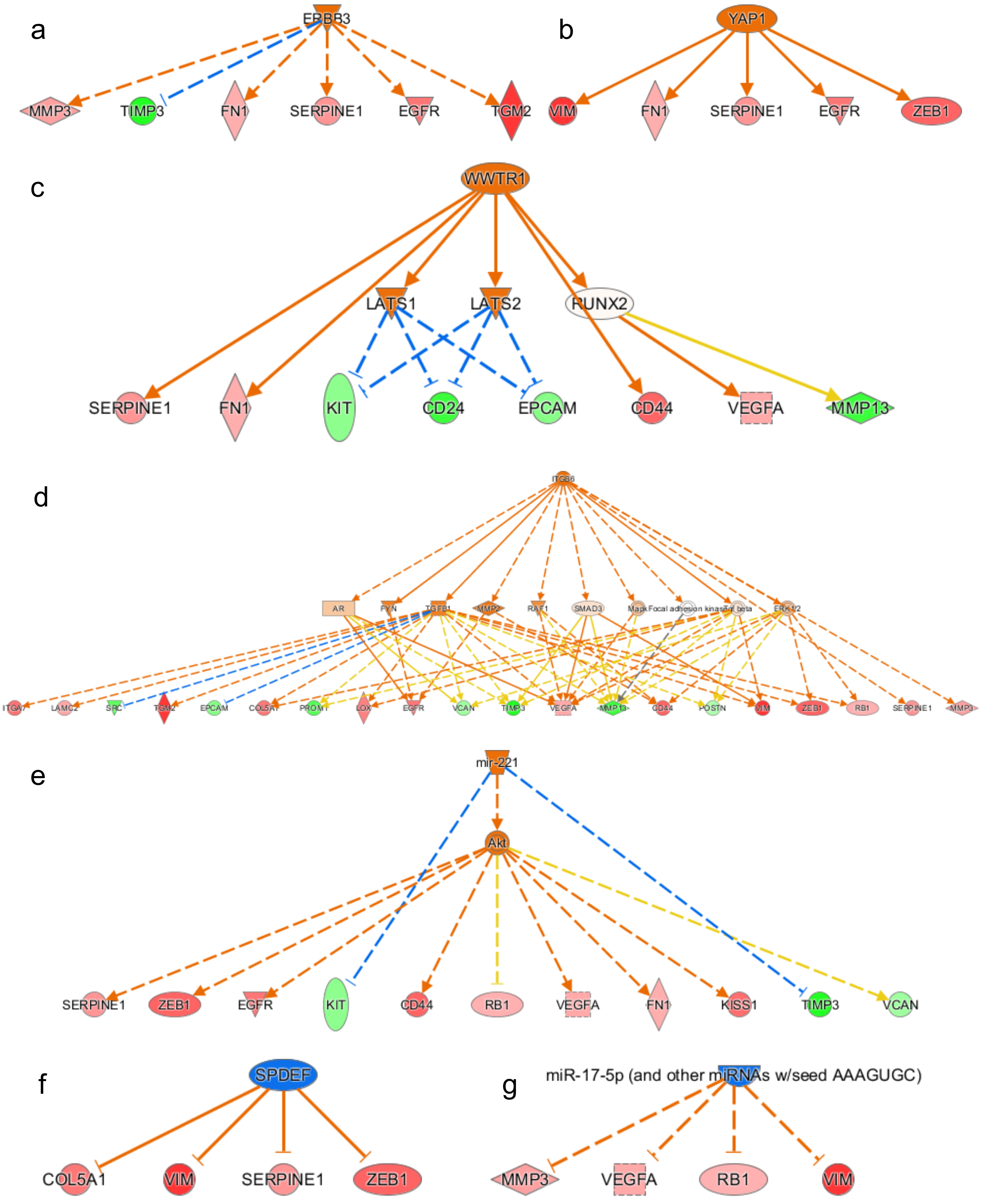

a
ERBB3
MMP3
TIMP3
FN1
SERPINE1
EGFR
TGM2
b
YAP1
VIM
FN1
SERPINE1
EGFR
ZEB1
c
WWTR1
LATS1
LATS2
RUNX2
SERPINE1
FN1
KIT
CD24
EPCAM
CD44
VEGFA
MMP13
d
ITGB6
AR
FYN
TGFB1
MMP2
RAF1
SMAD3
ERK1/2
ITGA7
LAMC2
SRC
TGM2
EPCAM
COL5A1
PROM1
LOX
EGFR
VCAN
TIMP3
VEGFA
MMP13
CD44
POSTN
VIM
ZEB1
RB1
SERPINE1
MMP3
e
mir-221
Akt
SERPINE1
ZEB1
EGFR
KIT
CD44
RB1
VEGFA
FN1
KISS1
TIMP3
VCAN
f
SPDEF
COL5A1
VIM
SERPINE1
ZEB1
g
miR-17-5p (and other miRNAs w/seed AAAGUGC)
MMP3
VEGFA
RB1
VIM

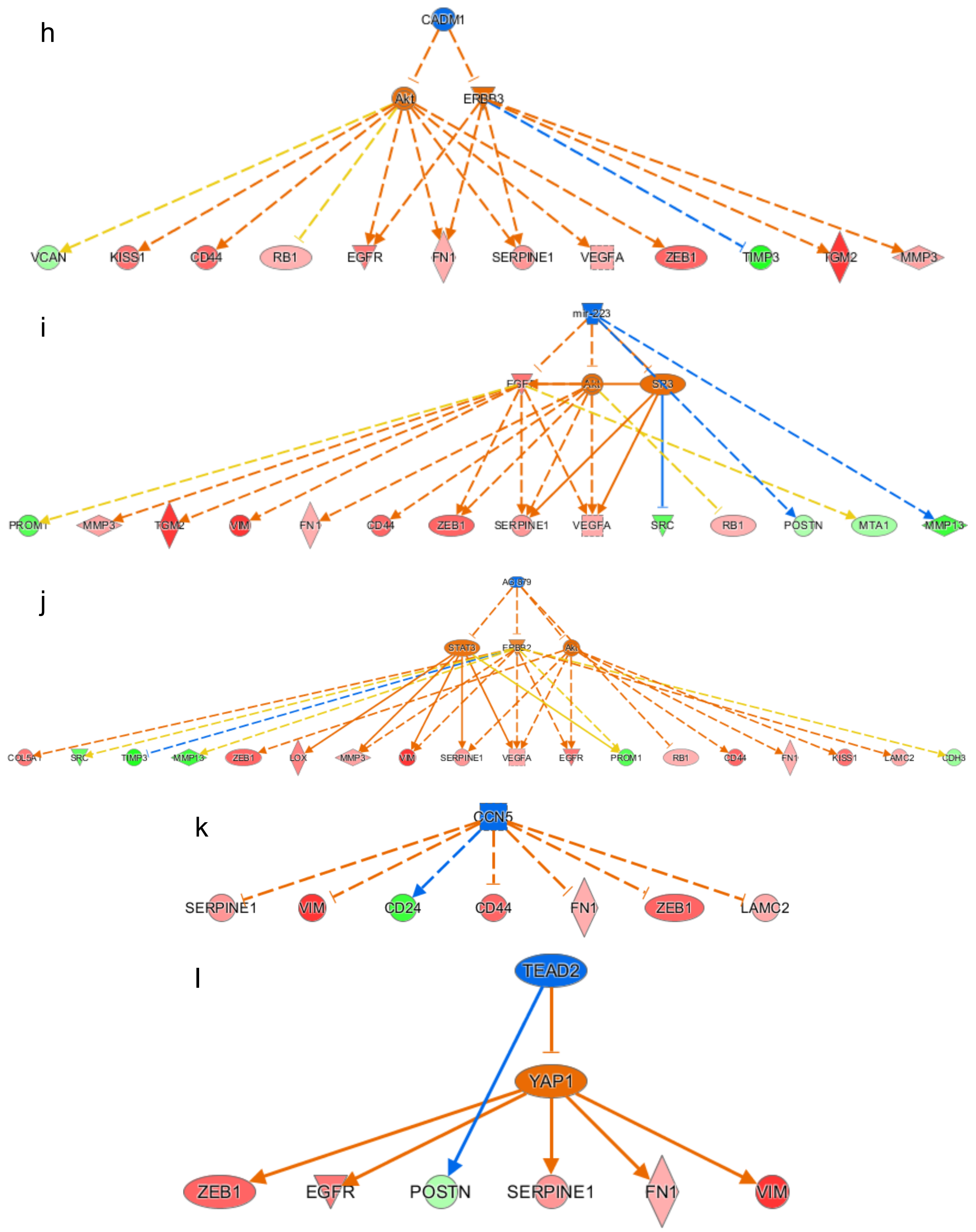
h
CADM1
Akt
ERBB3
VCAN
KISS1
CD44
RB1
EGFR
FN1
SERPINE1
VEGFA
ZEB1
TIMP3
TGM2
MMP3
i
mir-223
EGFR
Akt
SP3
PROM1
MMP3
TGM2
VIM
FN1
CD44
ZEB1
SERPINE1
VEGFA
SRC
RB1
POSTN
MTA1
MMP13
j
AG 879
STAT3
ERBB2
Akt
COL5A1
SRC
TIMP3
MMP13
ZEB1
LOX
MMP3
VIM
SERPINE1
VEGFA
EGFR
PROM1
RB1
CD44
FN1
KISS1
LAMC2
CDH3
k
CCN5
SERPINE1
VIM
CD24
CD44
FN1
ZEB1
LAMC2
l
TEAD2
YAP1
ZEB1
EGFR
POSTN
SERPINE1
FN1
VIM

m

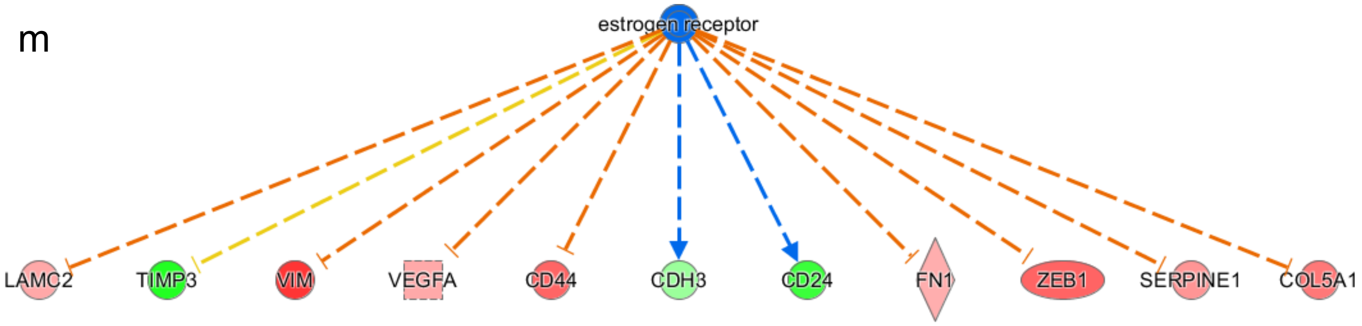

Supplementary Figure 3 - Network of genes predicted to be under the control of tau2 control nodes – a) *ERBB3,* b) *YAP1*, c) *WWTR1*, d) *ITGB6,* e) mir-221, f) *SPDEF*, g) miR-17-5p, h) *CADM1*, i) mir-223, j) AG879, k) *CCN5,* l) *TEAD2,* and m) estrogen receptor. Blue nodes represent regulators predicted to increase tau2. Orange nodes represent regulators predicted to decrease tau2. Green nodes are genes negatively correlated with tau2 and red nodes are genes positively correlated with tau2. Yellow lines represent relationships where a regulator predicted to increase tau2 actually increases the expression of genes negatively correlated with tau2 or decreases expression of positively correlated tau2 genes as well as the opposite for predicted tau2 inhibitor regulators. Dashed lines indicate indirect interactions while solid lines indicate direct interactions as demonstrated in previous literature.
