# Supplementary material for: Network analysis of gene expression reveals regulators of cell viscosity and mechanical phenotype
Source: Sci Rep. 2025 Sep 30;15:34008. doi: 10.1038/s41598-025-11698-0 (PMC12484610; doi:10.1038/s41598-025-11698-0)
Supplement: Supplementary file 1 — Supplementary Information. [file 41598_2025_11698_MOESM1_ESM.zip › FigureS4.pdf]

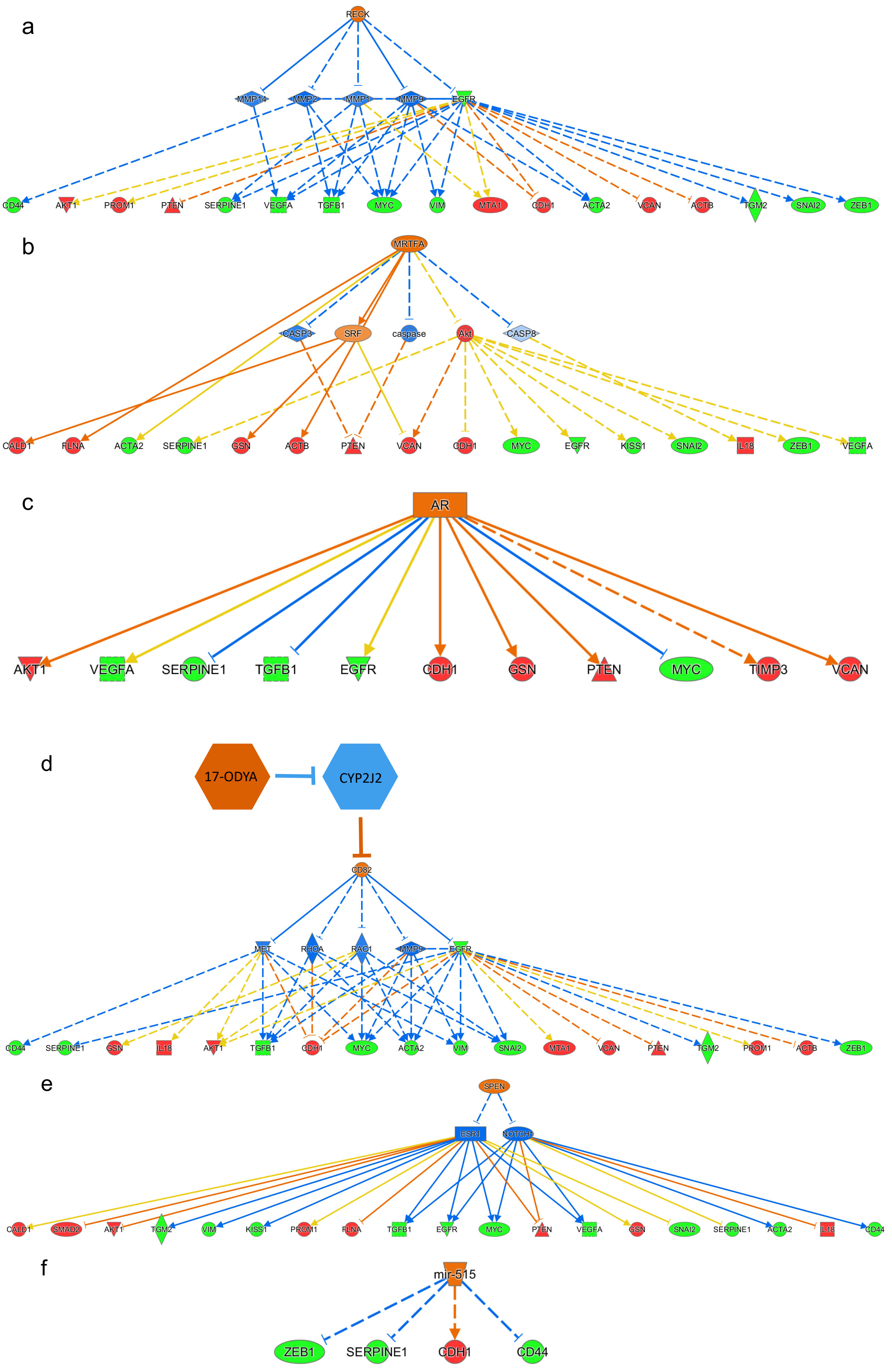
a
RECK
MMP14
MMP2
MMP1
MMP9
EGFR
CD44
AKT1
PROM1
PTEN
SERPINE1
VEGFA
TGFB1
MYC
VIM
MTA1
CDH1
ACTA2
VCAN
ACTB
TGM2
SNAI2
ZEB1
b
MRTFA
CASP3
SRF
caspase
Akt
CASP8
CALD1
FLNA
ACTA2
SERPINE1
GSN
ACTB
PTEN
VCAN
CDH1
MYC
EGFR
KISS1
SNAI2
IL18
ZEB1
VEGFA
c
AR
AKT1
VEGFA
SERPINE1
TGFB1
EGFR
CDH1
GSN
PTEN
MYC
TIMP3
VCAN
d
17-ODYA
CYP2J2
CD82
MET
RHOA
RAC1
MMP9
EGFR
CD44
SERPINE1
GSN
IL18
AKT1
TGFB1
CDH1
MYC
ACTA2
VIM
SNAI2
MTA1
VCAN
PTEN
TGM2
PROM1
ACTB
ZEB1
e
SPEN
ESR1
NOTCH1
CALD1
SMAD2
AKT1
TGM2
VIM
KISS1
PROM1
FLNA
TGFB1
EGFR
MYC
PTEN
VEGFA
GSN
SNAI2
SERPINE1
ACTA2
IL18
CD44
f
mir-515
ZEB1
SERPINE1
CDH1
CD44

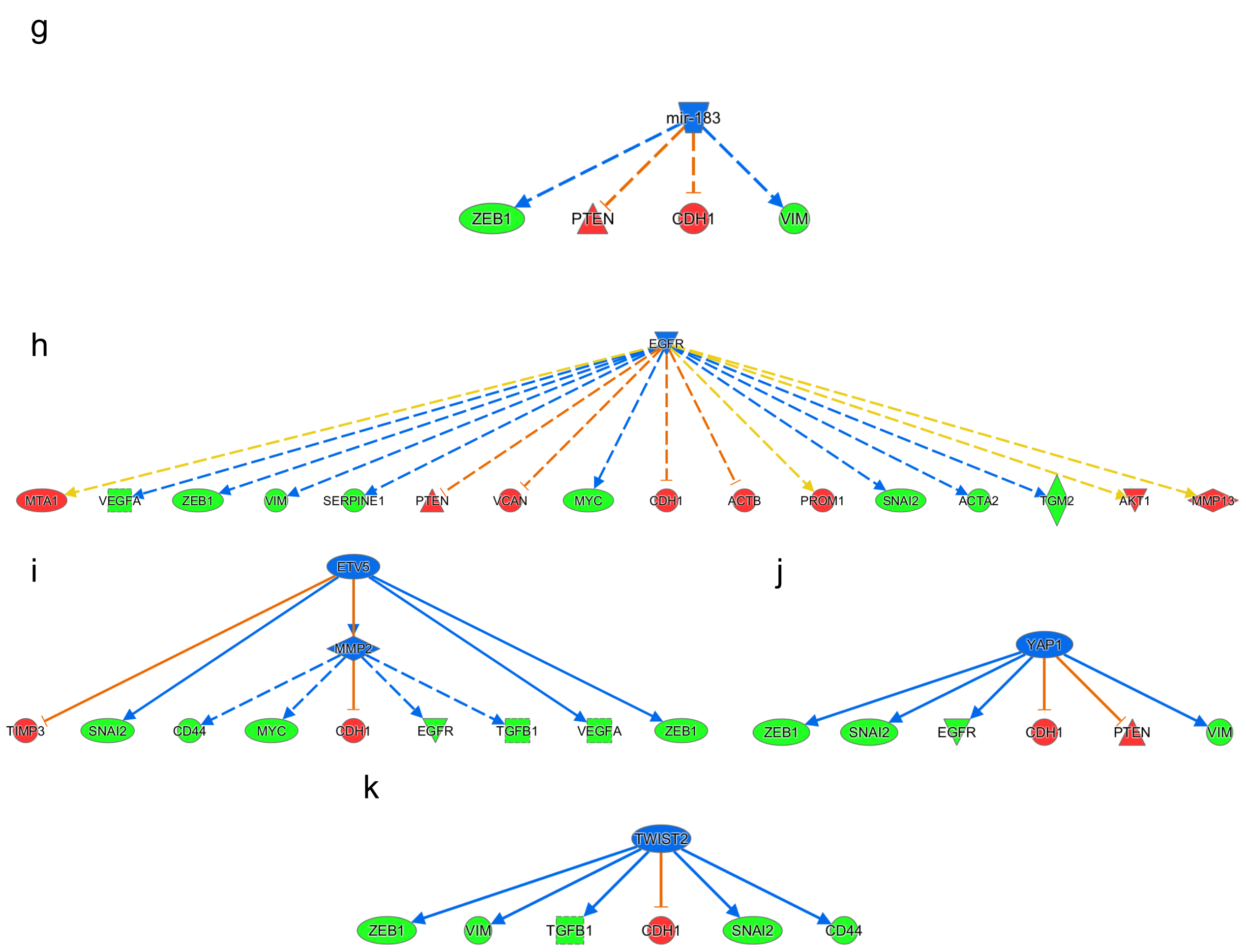


**Supplementary Figure 4 - Network of genes predicted to be under the control of stiffness control nodes –** Signal pathways for a) RECK, b) MRTFA, c) AR, d) CD82 and 17-ODYA activator, e) SPEN, f) miR515, g) miR183, h) EGFR, i) ETV5, j) YAP1 and k) TWIST2. Blue nodes represent regulators predicted to result in cell softening. Orange nodes represent regulators predicted to result in cell stiffening. Green nodes represent genes negatively correlated with cell stiffness and red nodes represent genes positively correlated with cell stiffness. Yellow lines represent relationships where a regulator is predicted to increase stiffness actually increases the expression of genes negatively correlated with stiffness or decreases expression of positively correlated stiffness genes as well as the opposite for predicted cell softening regulators. Dashed lines indicate indirect interactions while solid lines indicate direct interactions as demonstrated in previous literature.
