# Supplementary material for: Network analysis of gene expression reveals regulators of cell viscosity and mechanical phenotype
Source: Sci Rep. 2025 Sep 30;15:34008. doi: 10.1038/s41598-025-11698-0 (PMC12484610; doi:10.1038/s41598-025-11698-0)
Supplement: Supplementary file 1 — Supplementary Information. [file 41598_2025_11698_MOESM1_ESM.zip › TableS1.pdf]

Supplementary Table 1 – miR-183 expression levels after transfection – A miR-183 mimic and miR-183 inhibitor were used to overexpress and inhibit the expression of *miR-183* in three ovarian cancer cell lines. Fold expression is calculated with the $\Delta\Delta C_t$ method normalizing RT-qPCR C_t values to housekeeping gene *RNU6B* expression and *miR-183* levels of samples that were transfected with both the negative control miRNA (miRNC) mimic and inhibitor.

**Treatment and Cell Line**	**miR-183 Fold Change**		
**miR-183 mimic**	**+**	**-**	**+**
**miR-183 inhibitor**	**-**	**+**	**+**
HEY A8	41,866.400	0.777	13,312.299
HEY	620.540	0.312	578.150
OVCAR3	2,594.437	5.180	4,495.697
